# Lung Cancer Patients’ Characteristics and Comorbidities Using the Korean National Hospital Discharge In-depth Injury Survey Data

**DOI:** 10.1007/s44197-022-00044-6

**Published:** 2022-06-01

**Authors:** Kyunghee Lee, Sunghong Kang, Jieun Hwang

**Affiliations:** 1 Department of Healthcare Management, Eulji University, 553 Sanseongdae-ro, Sujeong-gu, Seongnam, Kyeonggi-do 13135 South Korea; 2 Department of Health Policy and Management, Inje University, 197 Inje-ro, Kimhae, Kyungsangnam-do 50834 South Korea; 3 College of Health Science, Dankook University, 119 Dandae-ro, Dongnam-gu, Cheonan, Chungcheongnam-do 31116 South Korea

**Keywords:** Lung cancer, Epidemiology, Chronic disease, Association rules

## Abstract

**Background:**

The aim of this study was to assess the incidence of lung cancer and comorbidities in Korea and analyze the lung cancer patient’s characteristics and their comorbidities over the past 12 years. This study also aimed to investigate factors related to death as treatment outcome in discharged lung cancer patients.

**Methods:**

This study analyzed the data obtained from the Korean National Hospital Discharge In-depth Injury Survey from 2006 to 2017. The quantity of discharged lung cancer patients was assessed by year. Comorbidities were limited to those included in the Elixhauser Comorbidity Index (ECI). A Chi-square test was performed to determine statistically significant differences in the distributions of the ECI and ECI scores according to the presence or absence of metastatic cancer. Logistic regression analysis was used to analyze factors related to death as treatment outcome.

**Results:**

From 2006 to 2017, the number of discharged male and female patients with lung cancer increased from 31,720 to 42,016 and 10,897 to 18,197, respectively. The increase in the number of lung cancer patients was greater in women than in men (67.0% vs. 32.5%, respectively). The most common associated comorbidities were hypertension, diabetes, and chronic pulmonary disease. The factors related to death as treatment outcome were found to include sex, admission route, number of hospital beds, length of stay, presence or absence of metastatic cancer, and ECI score.

**Conclusion:**

The number of lung cancer patients in Korea has increased, and a high proportion of these patients have chronic diseases, which negatively would impact the treatment and outcome of lung cancer patients as well as their quality of life. Thus, the management of chronic diseases needs to be prioritized in patients with lung cancer.

## Background

Currently, cancer is the leading cause of death in Korea, and the second leading cause of death worldwide [1]. As of 2020, the global incidence of death from cancer was 8.97 million people annually [1]; however, that number is expected to increase to 18.63 million by 2060 [2]. In particular, the disability-adjusted life years suggests that cancer has the highest therapeutic and socioeconomic burden among human diseases [1, 2].

Globally, lung cancer is one of the common cancers and causes the greatest number of deaths (1.8 million people), followed by breast cancer (2.26 million people), colorectal cancer (2.21 million people), and breast cancer (1.93 million people) [1]. The cancer death rate in Korea is highest in the order of lung cancer, liver cancer, colorectal cancer, stomach cancer, and pancreatic cancer [3]. Particularly, those over the age of 60 years have the highest lung cancer mortality, and the lung cancer death rate is increasing at a steady rate compared to that 10 years ago. In addition, the incidence of lung cancer continues to increase in Asian women, and the cause of such an increase is unknown [1, 2].

Lung cancer incidence and mortality are related to air pollution, radon, occupational exposure, and heredity. Smoking is the main cause of lung cancer [4]. Approximately 75% of lung cancers can be prevented by eliminating tobacco carcinogens [5]. As the smoking rate has been reduced and cigarette filters have been developed, the incidence of adenocarcinoma is increasing, while that of squamous cell carcinoma is decreasing worldwide [6]. However, the smoking rate of Korean men is higher than that of men in other major countries. Although the risk of death in smokers compared to that of non-smokers is lower in Korea than in other countries, lung cancer remains one of the leading causes of death in Korea [7]. Nevertheless, advancements in early detection methods using low-dose computed tomography screening, endobronchial ultrasound, navigational bronchoscopy, video-assisted thoracic surgery, external beam radiotherapy, stereotactic body radiotherapy, diagnostic procedures, and treatment have greatly improved the early detection, morbidity, and mortality of lung cancer.

Previously, cross-sectional studies have reported trends in the incidence, death, and survival of lung cancer. Park et al. [8] and Shin et al. [9] reported that both the lung cancer mortality and survival rates increased in Korea between 2008 and 2012. Similar trends have been observed in the United States, United Kingdom, Egypt, and India [10]. While comorbidities are not directly related to patients’ previous history of hospitalization, they have major effects on the outcome of complications, length of hospital stay, or survival of patients with lung cancer. However, the association between the incidence of lung cancer and comorbidities in Korea has not yet been addressed. The aim of this study was to assess the incidence of lung cancer and comorbidities in Korea and analyze the lung cancer patient’s characteristics and their comorbidities over the past 12 years. This study also aimed to investigate factors related to death as treatment outcome in discharged lung cancer patients.

## Materials and Methods

### Participants

This study analyzed patients discharged from 2006 to 2017 using data from the Korean National Hospital Discharge In-depth Injury Survey. Data were collected by the Korea Disease Control and Prevention Agency (KDCA) to establish cost-effective health policies at a national level by identifying time-series trends in the size and characteristics of discharged patients [11].

The target population comprised all patients discharged from general hospitals across the country, and the survey population was all those who were discharged from hospitals with more than 100 beds. A total of 170 hospitals nationwide were selected for the sample according to bed size and survey method [12]. This is equivalent to approximately 9% of the survey population. The data collected included information on sociodemographic characteristics (sex, age), admission route (emergency, outpatient), insurance type (national health insurance, Medicaid), treatment outcome (survival, death), number of hospital beds (100–299, 300–499, 500–999, ≥ 1000) and length of stay (≤ 7, 8–13, 14–20, ≥ 21).

In this study, patients with the main diagnosis of malignant neoplasms of the trachea, bronchi, and lung (C33–C34) were defined as lung cancer patients, according to the Korean Standard Classification of Diseases (KCD-7th). Moreover, the Korean National Hospital Discharge In-depth Injury Survey data included up to 20 cases of secondary diagnosis, which were defined as comorbidities. If there are duplicate patient identification numbers, only the initial case with the earliest admission date was defined as a sample and included for analysis. Comorbidities were limited to those included in the Elixhauser co-morbidity index (ECI). Compared to other indicators of comorbidities, ECI is considered to better control for confounding variables in cancer research. ECI is an output indicator that assesses the presence or absence of 31 selected diseases, including congestive heart failure, cardiac arrhythmia, valvular disease, pulmonary circulation disorders, peripheral vascular disorders, hypertension uncomplicated, hypertension complicated, paralysis, other neurological disorders, chronic pulmonary disease, diabetes uncomplicated, diabetes complicated, hypothyroidism, renal failure, liver disease, peptic ulcer disease excluding bleeding, AIDS, lymphoma, solid tumor with metastasis, solid tumor without metastasis, rheumatoid arthritis, coagulopathy, obesity, weight loss, fluid and electrolyte disorders, blood loss anemia, deficiency anemia, alcohol abuse, drug abuse, psychoses, depression. The coding of comorbidities using the ECI was based on a study by Quan et al. [13]. With regard to metastatic cancer, the participants were classified into discharged patients with metastatic lung cancer and discharged patients with non-metastatic lung cancer according to the presence or absence of solid tumor with metastasis as defined by the ECI.

### Research Model and Analysis Method

The trends of male and female lung cancer patients discharged by year were assessed. A two-stage stratified cluster sampling method was used to calculate the total number of discharged patients and the rate of discharge [11].

To assess the characteristics of the patients, frequency analyses of sex, age, admission route, insurance type, treatment outcome, number of hospital beds, and length of stay were conducted for each of the with/without metastatic cancer. A Chi-square test was used to determine significant differences in the distribution of the characteristics of the participants between the metastatic lung patient and non-metastatic lung cancer groups. In addition, the distribution of each co-morbidity between the metastatic lung cancer and non-metastatic lung cancer groups was investigated, and the ECI scores were calculated according to the number of comorbidities (0 co-morbidity = ECI score 0; 1–2 comorbidities = ECI score 1; 3–4 comorbidities = ECI score 2; 5 or more = ECI score 3). A Chi-square test was performed to determine whether there were statistically significant differences in the distribution of comorbidities according to the presence or absence of metastatic cancer.

Finally, logistic regression analysis was performed to identify factors related to death compared to survival as treatment outcomes, and the results were presented as adjusted odds ratio (aOR) and 95% confidence interval.

Statistical Analysis System 9.4 software was used to investigate the number and characteristics of discharged lung cancer patients. Statistical significance was set at *p* < 0.05.

## Results

### Number of Discharged Lung Cancer Patients and Discharge Rate by Year

**Fig. 1:**
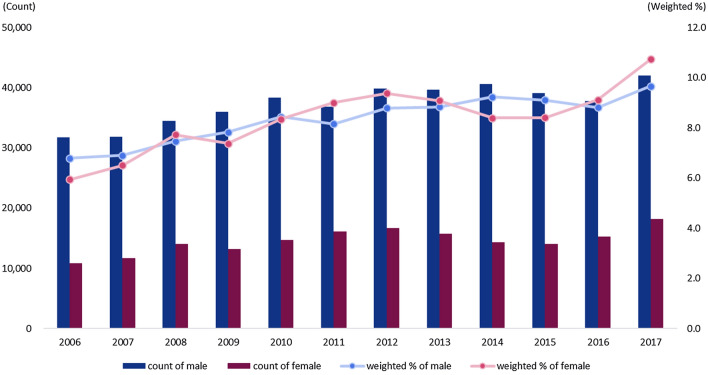
Number of discharged lung cancer patients and discharge rate (weighted) by sex (2006–2017)

The number of discharged lung cancer patients and discharge rates from 2006 to 2017 is shown in Fig. 1. In terms of the number of discharged lung cancer patients in 2006, there were 31,720 discharged male lung cancer patients and 10,897 discharged female lung cancer patients. The number of lung cancer patients by year repeatedly increased and decreased over the years. In 2017, there were 42,016 male lung cancer patients, and 18,197 female lung cancer patients. In terms of the number of discharged lung cancer patients by year, the number of discharged male lung cancer patients was higher compared to discharged female lung cancer patients. However, the rate of increase in the number of lung cancer patients in 2017 compared to 2006 was 32.5% in male lung cancer patients and 67.0% in female lung cancer patients, showing a higher increase in female lung cancer patients compared to male lung cancer patients.

In 2006, compared to the total discharge rate, the discharge rate of lung cancer patients was 5.9% in women, which was lower than that in men (6.8%). However, in 2017, the discharge rate of lung cancer patients increased to 10.7% in women, which was higher than the 9.7% in men.

### Characteristics of Lung Cancer Patients

The results of comparing sociographic characteristics of lung cancer patients according to the presence or absence of metastatic cancer are shown in Table 1.

**Table 1:** Characteristics of lung cancer patients with metastatic cancer

Variables	With metastatic cancer		With non-metastasis cancer		*p* value^†^
	*n*	*n*	%	%	
Sex					< 0.001
Men	7378	69.4	10,908	73.3	
Women	3259	30.6	3983	26.7	
Age					< 0.001
0–24	5	0.0	19	0.1	
25–44	434	4.1	343	2.3	
45–64	4278	40.2	4640	31.2	
≥ 65	5920	55.7	9889	66.4	
Admission route					< 0.001
Emergency	3444	32.4	3357	22.5	
Outpatient	7193	67.6	11,534	77.5	
Insurance type					< 0.001
NHI	9770	91.8	13,496	90.6	
Medicaid	867	8.2	1395	9.4	
Outcomes					< 0.001
Survival	8910	83.8	13,431	90.2	
Death	1727	16.2	1460	9.8	
Number of hospital beds					< 0.001
100–299	347	3.3	1668	11.2	
300–499	567	5.3	1369	9.2	
500–999	5567	52.3	8081	54.3	
≥ 1000	4156	39.1	3773	25.3	
Length of stay					< 0.001
≤ 7	4530	42.6	8036	54.0	
8–13	2746	25.8	3624	24.3	
14–20	1406	13.2	1488	10.0	
≥ 21	1955	18.4	1743	11.7	

*NHI* National Health Insurance

^†^Chi-square test

By gender, there were 7323 discharged male patients with metastatic lung cancer (69.4%) and 3259 discharged female patients with metastatic lung cancer (30.6%), and there were 10,908 discharged male patients with non-metastatic lung cancer (73.3%) and 3983 discharged female patients with non-metastatic lung cancer (26.7%). The proportion of male patients in both the metastatic lung cancer group and the non-metastatic lung cancer group was higher, but the proportion of female patients in the metastatic cancer lung cancer group was higher compared to the non-metastatic lung cancer group (*p* < 0.001).

In terms of the age distribution of discharged metastatic lung cancer patients, the numbers of those aged 0–24 years, 25–44 years, 45–64 years, and 65 years or older were 5 (0.0%), 434 (4.1%), 4278 (40.2%), and 5920 (55.7%), respectively. In terms of the age distribution of discharged non-metastatic lung cancer patients, the number of those aged 0–24 years, 25–44 years, 45–64 years, and 65 years or older was 19 (0.1%), 343 (2.3%), 4,640 (31.2%), and 9,889 (66.4%), respectively. The proportion of those aged 24–64 was higher in the metastatic lung cancer patients, whereas the proportion of those aged 65 years or older was high in the non-metastatic lung cancer group (p < 0.001).

In terms of admission route, 3444 (32.4%) discharged metastatic lung cancer patients were admitted to hospitals through the emergency department, while 3357 (22.5%) discharged non-metastatic lung cancer patients were admitted to hospitals through the emergency department, indicating that the proportion of metastatic lung cancer patients who were admitted via emergency department was higher. In terms of admission through outpatient department, 7193 (67.6%) discharged patients with metastatic lung cancer were admitted to hospitals through outpatient department, while 11,534 (77.5%) discharged patients with non-metastatic lung cancer were admitted to hospital through outpatient department, indicating that the proportion of non-metastatic lung cancer patients who were admitted to hospitals through outpatient department was higher (*p* < 0.001).

In terms of insurance type, the discharged metastatic lung cancer group consisted of 9770 (91.8%) national health insurance recipients, and 867 (8.2%) Medicaid recipients, while the discharged non-metastatic lung cancer group consisted of 13,496 (90.6%) national medical insurance recipients, and 1395 (9.4%) Medicaid recipients, indicating that the proportion of medical aid recipients was higher in the non-metastatic lung cancer group (*p* < 0.001).

The numbers of discharged patients with metastatic lung cancer and discharged patients with non-metastatic lung cancer whose treatment outcome was recorded as survival were 8910 (83.8%), and 13,431 (90.2%), respectively. Meanwhile, the numbers of discharged patients with metastatic lung cancer and those with non-metastatic lung cancer whose treatment outcome was recorded as death were 1727 (16.2%) and 1460 (9.8%), respectively, indicating that the incidence of deaths was higher in discharged patients with metastatic lung cancer (*p* < 0.001).

The discharged metastatic lung cancer group consisted of 347 patients (3.3%) discharged from hospitals with 100–299 beds, 567 patients (5.3%) from hospitals with 300–499 beds, and 5567 (52.3%) from hospitals with 500–999 beds, and 4,156 patients (39.1%) from hospitals with over 1000 beds. The discharged non-metastatic lung cancer group consisted of 1168 patients (11.2%) discharged from hospitals with 100–299 beds, 1369 patients (9.2%) from hospitals with 300–499, 8081 patients (54.3%) from hospitals with 500–999, and 3773 patients (25.3%) from hospitals with over 1000 beds. In terms of patient distribution according to the number of hospital beds, the proportion of those discharged from hospitals with 100–999 beds was lower in the discharged metastatic lung cancer group compared to the discharged non-metastatic lung cancer group, whereas the proportion of those discharged from hospitals with over 1000 beds was higher in the discharged metastatic lung cancer group compared to the discharged non-metastatic lung cancer group (*p* < 0.001).

In terms of the length of stay, the number of discharged patients with metastatic lung cancer whose length of stay was ≤ 7 days was 4530 (42.6%), whereas the number of those with non-metastatic lung cancer whose length of stay was ≤ 7 days was 8036 (54.0%), accounting for more than half of the participants. In addition, the number of discharged patients with metastatic lung cancer whose length of stay was ≥ 21 days was 1955 (18.4%), whereas the number of those with non-metastatic lung cancer was 1743 (11.7%), indicating that the proportion of metastatic lung cancer patients whose length of stay was ≥ 21 days was higher (*p* < 0.001).

### Comorbidities

The results of investigating the distribution of comorbidities according to the presence or absence of metastatic cancer are shown in Table 2.

**Table 2:** Distribution of comorbidities by Elixhauser Comorbidity Index with/without metastatic cancer

Diagnosis	With metastatic cancer		With non-metastasis cancer		*p* value^†^
	*n*	%	*n*	%	
Congestive heart failure	94	0.9	156	1.0	0.397
Cardiac arrhythmia	233	2.2	346	2.3	0.529
Valvular disease	32	0.3	43	0.3	0.751
Pulmonary circulation disorders	173	1.6	165	1.1	0.001
Peripheral vascular disorders	52	0.5	83	0.6	0.732
Hypertension uncomplicated	2,645	24.9	3,365	22.6	< .001
Hypertension complicated	35	0.3	69	0.5	0.097
Paralysis	47	0.4	27	0.2	< .001
Other neurological disorders	129	1.2	76	0.5	< .001
Chronic pulmonary disease	708	6.7	1,619	10.9	< .001
Diabetes uncomplicated	1,488	14.0	1,998	13.4	0.110
Diabetes complicated	89	0.8	139	0.9	0.787
Hypothyroidism	95	0.9	113	0.8	0.239
Renal failure	130	1.2	212	1.4	0.474
Liver disease	211	2.0	273	1.8	0.828
Peptic ulcer disease excluding bleeding	96	0.9	122	0.8	0.455
AIDS	0	0.0	1	0.0	0.398
Lymphoma	10	0.1	16	0.1	0.740
Solid tumor without metastasis	590	5.5	801	5.4	0.252
Rheumatoid arthritis	40	0.4	69	0.5	0.493
Coagulopathy	68	0.6	84	0.6	0.491
Obesity	0	0.0	0	0.0	0.000
Weight loss	16	0.2	44	0.3	0.018
Fluid and electrolyte disorders	168	1.6	130	0.9	< .001
Blood loss anemia	2	0.0	3	0.0	0.940
Deficiency anemia	32	0.3	52	0.3	0.506
Alcohol abuse	45	0.4	84	0.6	0.239
Drug abuse	2	0.0	1	0.0	0.380
Psychoses	15	0.1	17	0.1	0.550
Depression	99	0.9	97	0.7	0.035
ECI score					< .001
0	0	0.0	8,252	55.4	
1	6,299	59.2	5,772	38.8	
2	3,301	31.0	790	5.3	
3	1,037	9.7	77	0.5	

^†^Chi-square test

The most common co-morbidity in discharged lung cancer patients was hypertension uncomplicated [those with metastatic cancer: 2645/24.9% vs. those with non-metastatic cancer: 3365 (22.6%)], followed by diabetes uncomplicated (those with metastatic cancer: 1488/14.0% vs. those with non-metastatic cancer: 1998/13.4%), chronic pulmonary disease (those with metastatic cancer: 708/6.7% vs. those with non-metastatic cancer: 1619/10.9%), solid tumor without metastasis (those with metastatic cancer: 590/5.5% vs. those with non-metastatic cancer: 801/5.4%), and cardiac arrhythmia (those with metastatic cancer: 233/2.2% vs. those with non-metastatic cancer: 346/2.3%).

Comorbidities that occurred at a higher incidence in patients with metastatic cancer compared to those with non-metastatic cancer included pulmonary circulation disorders (those with metastatic cancer: 173/1.6% vs. those with non-metastatic cancer: 165/1.1%, *p* < 0.001), hypertension (those with metastatic cancer: 2645/24.9% vs. those with non-metastatic cancer: 3365/22.6%, *p* < 0.001), paralysis (those with metastatic cancer: 47/0.4% vs. those with non-metastatic cancer: 27/0.2%, *p* < 0.001), other neurological disorders (those with metastatic cancer: 129/1.2% vs. those with non-metastatic cancer: 76/0.5%, *p* < 0.001), fluid and electrolyte disorders (those with metastatic cancer: 168/1.6%) vs. those with non-metastatic cancer: 130/0.9%, *p* < 0.001), and depression (those with metastatic cancer: 99/0.9% vs. those with non-metastatic cancer: 97/0.7%, *p* = 0.035). Meanwhile, the incidence rates of chronic pulmonary diseases (those with metastatic cancer: 708/6.7% vs. those with non-metastatic cancer: 1,619/ 10.9%, *p* < 0.001), weight loss (those with metastatic cancer: 16/0.2% vs. those t metastatic cancer: 30/0.9%, *p* < 0.001) were higher in those without metastasis cancer.

The results of comparing the ECI scores according to the presence or absence of metastatic cancer revealed that the proportion of patients with metastatic lung cancer who had an ECI score of two or more points higher compared to patients with non-metastatic lung cancer (*p* < 0.001).

### Treatment Outcome

Table 3 shows the results of logistic regression analysis to examine factors related to death compared to survival in terms of treatment outcome. The mortality rate was 1.32 times higher in female lung cancer patients compared to male lung cancer patients (aOR 1.32, 95% CI 1.20–1.45). When examining the mortality rate in lung cancer patients by age group based on those aged 0–24 years, the results revealed that the mortality rate increased with increasing age, but this was not statistically significant (*p* > 0.05). Patients admitted through the emergency department had a 4.17 times higher mortality rate (aOR 4.17, 95% CI 3.85–4.53) compared to those admitted through outpatient department. However, there was no statistically significant association between insurance type and mortality in discharged patients. When looking at mortality rates in discharged lung cancer patients with lung cancer compared to those discharged from hospitals with 1000 beds, the risk of mortality in discharged lung cancer patients increased as the number of hospital beds decreased. Compared to those discharged from hospitals with 1000 beds, the risk of mortality in those discharged from hospitals with 500–999, those from hospitals with 300–499 beds, those from hospitals with 300–499 beds, and those from hospitals with 100–299 beds was 1.59 times higher (aOR 1.59, 95% CI 1.43–1.77), 2.84 times higher (aOR 2.84, 95% CI 2.43–3.32), and 3.92 times higher (aOR 3.92, 95% CI 3.38–4.55), respectively. In addition, prolonged length of stay was associated with an increased risk of mortality. Compared to those with a length of stay of 7 days, those with a length of stay of 14–15 days had a 1.72 times higher risk of mortality (aOR 1.72, 95% CI 1.52–1.95), and those with a length of stay of ≥ 21 days had a 3.64 times higher risk of mortality (aOR 3.64, 95% CI 3.29–4.04). Those with metastatic lung cancer had a 1.80 times higher risk of mortality (aOR 1.80, 95% CI 1.61–2.01) compared to those with non-metastatic cancer. Compared to discharged lung cancer patients with an ECI score of 1 point, those with an ECI score of 2 points had a 1.23 times increased risk of mortality (aOR 1.23, 95% CI 1.10–1.37), and those with an ECI score of 3 points had a 1.56 times increased risk of mortality (aOR 1.56, 95% CI 1.32–1.85).

**Table 3:** The results of logistic regression analysis to examine factors related to death

	aOR	95% CI	*p* value
Sex			
Men	Ref		
Women	1.32	1.20–1.45	< 0.0001
Age			
0–24	Ref		
25–44	1.72	0.21–13.90	0.9828
45–64	2.11	0.27–16.81	0.4293
≥ 65	2.35	0.30–18.70	0.233
Admission route			
Emergency	4.17	3.85–4.53	< 0.0001
Outpatient	Ref		
Insurance type			
NHI	Ref		
Medicaid	0.97	0.85–1.11	0.6287
Number of hospital beds			
100–299	3.92	3.38–4.55	< 0.0001
300–499	2.84	2.43–3.32	< 0.0001
500–999	1.59	1.43–1.77	< 0.0001
≥ 1000	Ref		
Length of stay			
≤ 7	Ref		
8–13	1.06	0.95–1.18	0.1106
14–20	1.72	1.52–1.95	< 0.0001
≥ 21	3.64	3.29–4.04	< 0.0001
Metastatic cancer			
No	Ref		
Yes	1.80	1.61–2.01	< 0.0001
ECI score			
0	1.17	1.04–1.32	0.412
1	Ref		
2	1.23	1.10–1.37	0.954
3	1.56	1.32–1.85	< 0.0001

*aOR* adjusted odds ratio, *CI* confidence interval, *NHI* National Health Insurance

## Discussion

In this study, we observed that the number and rate of discharged lung cancer patients increased every year in Korea. In particular, the rate of increase was greater in women than in men. Actually, lung cancer was the most commonly diagnosed cancer both men and women aged ≥ 65 years [14]. The aging of discharged lung cancer patients would have led to an increase in comorbidities, including not only metastatic cancer, but also hypertension and diabetes.

As smoking is the main cause of lung cancer [4, 15], the relationship between changes in smoking rates and trends in lung cancer is often assessed. As tobacco regulations have been enforced worldwide, the lung cancer death rate is decreasing [16]. In the United States, the incidence of lung cancer has continued to decrease since the 1960s, following a decrease in smoking rates [17]. This decreasing trend in lung cancer incidence and mortality is expected to continue until 2065 [18]. Similarly, in Australia, lung cancer mortality is expected to decrease continuously [19]. In the UK, although there are regional differences, lung cancer mortality has continued to decrease since 1991 [20]. In Korea, the smoking rate of men was 66.7% as of 1995, which was the highest among Organization For Economic Co-operation and Development (OECD) member countries at that time [16]. That rate has continued to decrease through the promotion of various tobacco control policies, and in 2018, it was reduced to 30.5% [16].

Among the OECD member countries, the smoking rate in Korean women is reported to be approximately 3–7%, and significantly lower than that of men [16]. However, despite the low smoking rate, the number of female patients with lung cancer continues to increase. In particular, the rate of increase is higher in women than in men. This increase in female lung cancer patients may be attributed to the low self-reporting of smoking in Asian culture, which would undermine the smoking rate in women [21]. Moreover, the high smoking rate in men may frequently expose women to second-hand smoke, which can affect the incidence of lung cancer. In fact, adenocarcinoma is a common morphological finding in women and non-smokers. These findings indicate the effects of second-hand smoke exposure. In addition, the vulnerable working environment of women, with their continuous exposure to air pollution, radon, carbon dioxide, and second-hand smoke, may increase the incidence of lung cancer in women. As a result, the number of female patients with lung cancer has not reached its peak, and women-centered smoking cessation programs and improvement of working environments are needed.

As the smoking rate has decreased, lung cancer mortality should also decrease in Korea. In other words, the effects of smoking are observed after 20–30 years. Thus, if the smoking rate is decreasing, the incidence of lung cancer must also decrease [9]. However, as shown in this study, the incidence of lung cancer has continued to increase, indicating that the incidence of lung cancer in Korea due to smoking has not reached its peak. This finding suggests that the screening of lung cancer patients is fundamental until the peak of lung cancer incidence is reached. In fact, late screening for lung cancer has direct negative effects on treatment outcomes and the survival rate of patients [22]. In 2019, lung cancer was added as a target cancer for national cancer screening tests in Korea for early activation and improved survival rates [22]. The target population of this national cancer screening test included high-risk men and women between the ages of 54 and 74 years who smoked for more than 30 years, and these individuals must be screened every 2 years. Although it is too early to assess the effects of the new system, pilot projects have demonstrated the feasibility and potential performance of the screening tests. An increase in the number of screening subjects is expected to allow early screening of patients with lung cancer. Thus, it is necessary to evaluate the effects of screening tests with continuous monitoring in future [22].

As shown in this study, discharged patients with lung cancer were more likely to have chronic diseases, such as hypertension and diabetes. However, only a limited number of studies have shown that these chronic diseases are common in patients with lung cancer [23, 24]. In diabetes, the effects of insulin exposure are sometimes considered a risk factor for lung cancer; however, the effects are not consistent in men and women [25]. Simultaneous diagnosis of hypertension in lung cancer patients with no chronic lung diseases, such as chronic obstructive pulmonary disease, is associated with a decreased survival rate, but the mechanism is still not clearly established [26]. As a result, chronic disease could not only negatively affect the treatment outcome of lung cancer patients, but also directly affect their quality of life [27, 28]. Thus, the management of chronic diseases is required for the treatment and management of patients with lung cancer.

In addition, this study found that there were differences in sex, age, admission route, insurance type, treatment outcome, number of beds at the time of discharge, and length of stay between the discharged metastatic lung cancer group and the discharged non-metastatic lung cancer group. This study also found that there were differences in the type and number of some comorbidities between the two groups. Eventually, we found that the presence of metastatic cancer was significantly associated with death as treatment outcome. In fact, it is known that lung cancer frequently recurs or metastasizes to other sites compared to other carcinomas [30]. In common cases, primary cancers can spread via the bloodstream to the lungs, leading to metastatic lung cancer. In general, such metastatic cancer is difficult to treat, and it may also imply that metastatic cancer patients are difficult to survive [31]. As found in this study, there were difference in treatment outcome, and the type and number of comorbidities according to the presence of metastatic cancer, which negatively affect treatment outcome. Therefore, therapeutic approaches that consider metastatic cancer along with chronic disease management are needed to improve survival and treatment outcome in lung cancer patients.

This study is meaningful because the number of discharged lung cancer patients in Korea was assessed over an extended period. In addition, changes in the sociodemographic characteristics of patients with lung cancer were assessed over time. It is also significant as comorbidities, which have not been assessed in other studies, were investigated. However, several limitations must be considered when interpreting the findings of this study. First, lung cancer patients were defined as those with the main diagnosis of lung cancer, and those patients with a lung cancer diagnosis in 20 different secondary diagnoses were not included in this study. Thus, the number of discharged patients with lung cancer may have been underestimated. Second, association rules have limitations in proving the precedence relationship between comorbidities. It is necessary to prove the mechanism of disease development in future studies. Third, only the disease outcomes of patients were analyzed. Other risk factors, such as smoking, drinking, and physical activities, were not considered, and these limitations need to be considered when interpreting our findings.

## Data Availability

The datasets analyzed during the current study are available from the Korea Disease Control and Prevention Agency on reasonable request [http://www.kdca.go.kr/contents.es?mid=a20303010502].

## Abbreviation

ECI: Elixhauser co-morbidity index
